# Supplementary material for: The Carrot Phytoene Synthase 2 (DcPSY2) Promotes Salt Stress Tolerance through a Positive Regulation of Abscisic Acid and Abiotic-Related Genes in Nicotiana tabacum
Source: Plants (Basel). 2023 May 9;12(10):1925. doi: 10.3390/plants12101925 (PMC10220825; doi:10.3390/plants12101925)
Supplement: Supplementary file 1 [file plants-12-01925-s001.zip › plants-2299367-supplementary.pdf]

Supplementary Figures

>gb|DQ192187.1|:357-1673 Daucus carota subsp. sativus putative phytoene synthase (PSY2) mRNA, complete cds

ATGTCAGTTGCTATGTCCTGGATTGTTACTCCCAGTCTTGAGGTTTCCAATTGCTTCGGGTACTTGGAGA

CTGCCCGAGAGGGAACCCGAGTATTAGATCCATCTAGGTTGGGTTCCCGGGATAAGAATATGAGGTGTGG

AGGCAGACTTGAGAAGGGTAAGCTGCGGAAGTGGAGTTCTAAATCTTTCAATGCTGAATATAGCTATTCG

TGTTTGGGTGGTTCTGAATTAGAGAATGGAAGTATATTTCCTGTGCATTCAAGTATGGTAGTTAGTGCAG

ATGGAGATATGGCAGTGTCATCAGAGAAAAAGGTATATGACGTGGTTCTTAAACAAGCAGCGTTGGTCAA

AAGACAGTTCAGATCTGATGAGGAATTAGAGGTTAAGCCAGAAATGATTCTTCCGGGGACTCTGAGCTTG

TTAAGTGAAGCTTATGATCGATGCGGTGAAGTATGTGCTGAGTATGCCAAAACATTTTACTTGGGAACAC

TACTGATGACCCCAGAGAGGCGGAGGGCTATCTGGGCAATATATGTGTGGTGCAGAAGAACTGATGAATT

GGTAGATGGACCTAATGCGTCACATATAACTCCTTCAGCTTTGGATAGGTGGGAGTTGAGATTAGAAGAT

CTTTTCAAGGGGCGTCCATTTGATATGCTTGATGCTGCTTTATCAGATACAGTAATGAAGTTTCCTGTTG

ACATCCAACCATTCAAAGATATGATTGAAGGGATGAGGATGGACCTTAAGAAGTCGAGATACAAAAACTT

CGACGAGCTATATCTTTATTGCTATTATGTTGCTGGTACTGTTGGATTGATGAGCGTTCCAATTATGGGC

ATTGCACCTAATTCACAGGCAACAACGGAGAGTGTTTATAATGCTGCTTTGGCTTTAGGGCTTGCTAATC

AATTGACTAACATACTCAGGGATGTTGGAGAAGATGCCAGAAGAGGAAGGGTTTATCTACCACAAGATGA

ACTGGCTCAAGCAGGGCTTTCTGATGAAGATATATTTGCGGGGAAGGTTACCGATAAATGGAGGAATTTC

ATGAAGAAGCAAATTAAGAGGGCAAGGATGTTTTTCGATGAAGCACAAATAGGAGTAAGAGAACTCAGCC

CAGCTAGTAGATGGCCGGTATGGGCATCACTGCTGTTGTACCGTCAAATACTAGATGAAATTGAAGCCAA

CGATTACAATAATTTTACGAAGAGGGCTTATGTCAGCAAACCCAAGAAAATACTTGCTTTGCCAGTTGCA

TATGCAAAAGCTTTTGCTCCAACAGCAAGAACAAGTTCAACGTTTCTGAAAACA**TGA**

Supplementary Figure 1. *DcPSY2* cloned sequence. The coding sequence of *DcPSY2* (1276 bp) was amplified and cloned into pCR8® entry vector. Start and stop codons are highlighted in bold.

```
Query  184   TCTTTCAATGCTGAATATAGCTATTCGTGTTTGGGTGGTTC-TGAATTAGAGAATGGAAG  242
             ||||| | ||||||    || ||||| || |||||||| ||  ||| | || || |||||
Sbjct  190   TCTTTAATTGCTGATCCAAGATATTCATGCTTGGGTGGATCAAGAACT-GAAAAGGGAAG  248

Query  243   TATATTTC-CTGTGCATTCAAGTATGGTAGTTAGTGCAGATGGAGATATGGCAGTGTCAT  301
              |  |||| |||| || || ||| |||| | |||  ||| |||||| ||| |||||||||
Sbjct  249   CA-GTTTCTCTGTACAGTCCAGTTTGGTGGCTAGCCCAGCTGGAGAAATGACAGTGTCAT  307

Query  302   CAGAGAAAAAGGTATATGACGTGGTTCTTAAACAAGCAGCGTTGGTCAAAAGACAGTTCA  361
             ||||||||||||| ||||| |||||  | || ||||||||| || || || || ||| | |
Sbjct  308   CAGAGAAAAAGGTCTATGATGTGGTATTGAAGCAAGCAGCTTTAGTGAAGAGGCAGCTGA  367

Query  362   GATCTGATGAGGAATTAGAGGTTAAGCCAGAAATGATTCTTCCGGGGACTCTGAGCTTGT  421
             |||||   || |||||||| || || || || ||  || |||| |||| | || ||||||
Sbjct  368   GATCTACCGATGAATTAGAAGTGAAACCTGATATAGTTGTTCCAGGGAATTTGGGCTTGT  427

Query  422   TAAGTGAAGCTTATGATCGATGCGGTGAAGTATGTGCTGAGTATGCCAAAACATTTTACT  481
             | |||||||| |||||||| || || |||||||||||| |||||||| || ||||||||||
Sbjct  428   TGAGTGAAGCATATGATCGTTGTGGCGAAGTATGTGCAGAGTATGCAAAGACATTTTACT  487

Query  482   TGGGAACACTACTGATGACCCCAGAGAGGCGGAGGGCTATCTGGGCAATATATGTGTGGT  541
             | ||||||   || ||||| ||||||||  | || ||||||||||||||||||||||||||
Sbjct  488   TAGGAACAAAGCTAATGACTCCAGAGAGAAGAAGAGCTATCTGGGCAATATATGTGTGGT  547

Query  542   GCAGAAGAACTGATGAATTGGTAGATGGACCTAATGCGTCACATATAACTCCTTCAGCTT  601
             |||| ||||| |||||  | || ||||| ||||| || ||||| ||||||||   |||||
Sbjct  548   GCAGGAGAACGGATGAGCTAGTCGATGGCCCTAACGCATCACACATAACTCCACAAGCTT  607

Query  602   TGGATAGGTGGGAGTTGAGATTAGAAGATCTTTTCAAGGGGCGTCCATTTGATATGCTTG  661
             | || |||||||||   ||  | |||||| ||||||  ||||| ||||||||||||||||
Sbjct  608   TAGACAGGTGGGAGGCCAGGCTGGAAGATATTTTCAGTGGGCGGCCATTTGATATGCTTG  667

Query  662   ATGCTGCTTTATCAGATACAGTAATGAAG-TTTCCTGTTGACATCCAACCATTCAAAGAT  720
             ||||||||||||| ||||| ||  |  || ||||||||||| || || ||||||| ||||
Sbjct  668   ATGCTGCTTTATCCGATACTGTC-TCCAGATTTCCTGTTGATATTCAGCCATTCAGAGAT  726

Query  721   ATGATTGAAGGGATGAGGATGGACCTTAAGAAGTCGAGATACAAAAACTTCGACGAGCTA  780
             ||||| ||||| ||| | ||||||| |   ||| || |||||| ||| | |||||| ||||||
Sbjct  727   ATGATAGAAGGAATGCGTATGGACTTGTGGAAATCCAGATATAACAACTTCGATGAGCTA  786

Query  781   TATCTTTATTGCTATTATGTTGCTGGTACTGTTGGATTGATGAGCGTTCCAATTATGGGC  840
             ||||| ||||| ||||||||||||||||||| || ||| |||||||| ||||||| ||||||| 
Sbjct  787   TATCTCTATTGTTATTATGTTGCTGGTACAGTAGGACTGATGAGTGTTCCAGTTATGGGT  846

Query  841   ATTGCACCTAATTCACAGGCAACAACGGAGAGTGTTTATAATGCTGCTTTGGCTTTAGGG  900
             ||||||||| | ||| |||||||||| |||||||| ||||||||||||||||||||||||
Sbjct  847   ATTGCACCTGAATCAAAGGCAACAACAGAGAGTGTATATAATGCTGCTTTGGCTTTAGGG  906

Query  901   CTTGCTAATCAATTGACTAACATACTCAGGGATGTTGGAGAAGATGCCAGAAGAGGAAGG  960
             ||||| |||||| | || ||  |||||||| ||||| ||||||||||||||||||||| | 
Sbjct  907   CTTGCAAATCAACTAACCAATATACTCAGAGATGTAGGAGAAGATGCCAGAAGAGGACGA  966

Query  961   GTTTATCTACCACAAGATGAACTGGCTCAAGCAGGGCTTTCTGATGAAGATATATTTGCG  1020
             || ||  |||| ||||||||| | || || ||||||||||||||||||||||||||||| 
Sbjct  967   GTATACTTACCTCAAGATGAATTAGCACAGGCAGGGCTTTCTGATGAAGATATATTTGCT  1026

Query  1021  GGGAAG-GTTACCGATAAATGGAGGAATTTCATGAAGAAGCAAATTAAGAGGGCAAGGAT  1079
             || ||| || |||||||| ||||||| ||| |||||||| |||||| ||||||| |||| 
Sbjct  1027  GG-AAGAGTGACCGATAAGTGGAGGAACTTTATGAAGAAACAAATTCAGAGGGCGAGGAA  1085

Query  1080  GTTTTTCGATGAAGCACAAATAGGAGTAAGAGAACTCAGCCCAGCTAGTAGATGGCCGGT  1139
              || || |||||  || | | ||| || | ||||||   | | |||||||||||||| ||
Sbjct  1086  ATTCTTTGATGAGTCAGAGAAAGGTGTCACAGAACTGGACTCTGCTAGTAGATGGCCTGT  1145

Query  1140  ATGGGCATCACTGCTGTTGTACCGTCAA-ATACTAGATGAAATTGAAGCCAACGATTACA  1198
              |  ||| | ||||||||||| || ||| ||| | || || ||||||||||| || ||||
Sbjct  1146  GTTAGCAGCGCTGCTGTTGTATCG-CAAGATATTGGACGAGATTGAAGCCAATGACTACA  1204

Query  1199  ATAATTTTACGAAGAGGGCTTATGTCAGCAAACCCAAGAAAATACTTGCTTTGCCAGTTG  1258
             |||| || || | ||||||||||||| ||||| || |||||  | ||  | |||||  |||
Sbjct  1205  ATAACTTCACAAGGAGGGCTTATGTTAGCAAGCCAAAGAAGCTTCTCACCTTGCCCATTG  1264

Query  1259  CATATGCAAAAGCTTTTG  1276
             | ||||||||| || |||
Sbjct  1265  CTTATGCAAAATCTCTTG  1282
```

Supplementary Figure 2. ***DcPSY2* and *NtPSY2* alignment.** The *DcPSY2* coding sequence (subject, 184bp-1276bp) presents 80% identity with *NtPSY2* (query, 190-1282bp; LOC107761716; XM 016579975.1). Score: 817 bits (442)

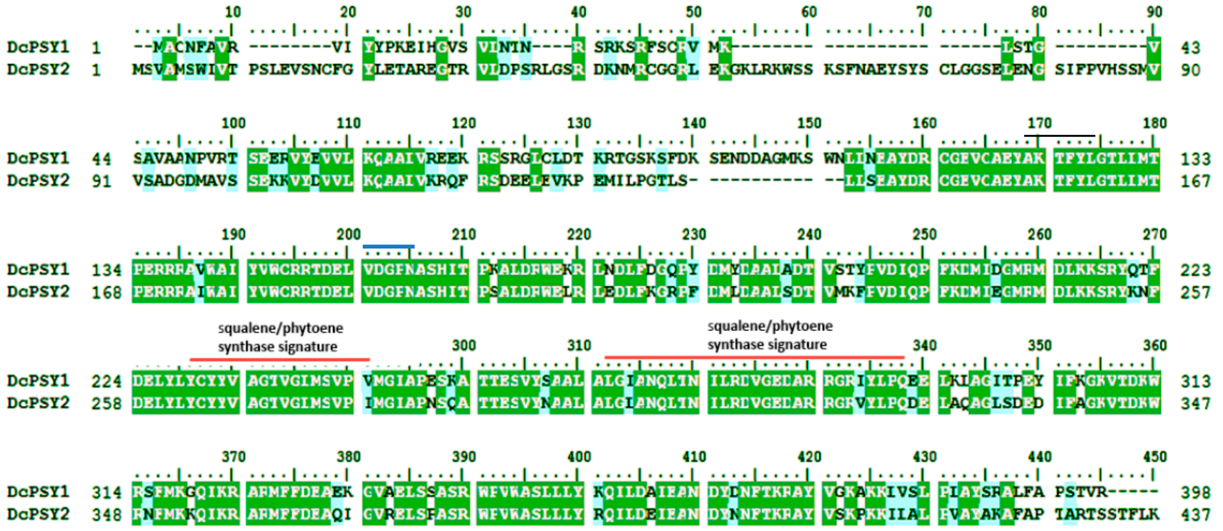

Supplementary Figure 3. DcPSY1 and DcPSY2 alignment. The predicted proteins DcPSY1 (398 aa) and DcPSY2 (437 aa) share 64% identity and present the same amino acids at the active site (highlighted in dark), Mg+2 binding site (highlighted in blue). Two squalene/phytoene synthase signatures are present in both *D. carota* PSY proteins (in red). Identical amino acids are highlighted in green. Similar amino acids are highlighted in light blue. The squalene/phytoene synthase, isoprenoid synthase and the trans-isoprenyl diphosphate synthase domains are depicted in Figure 2A.

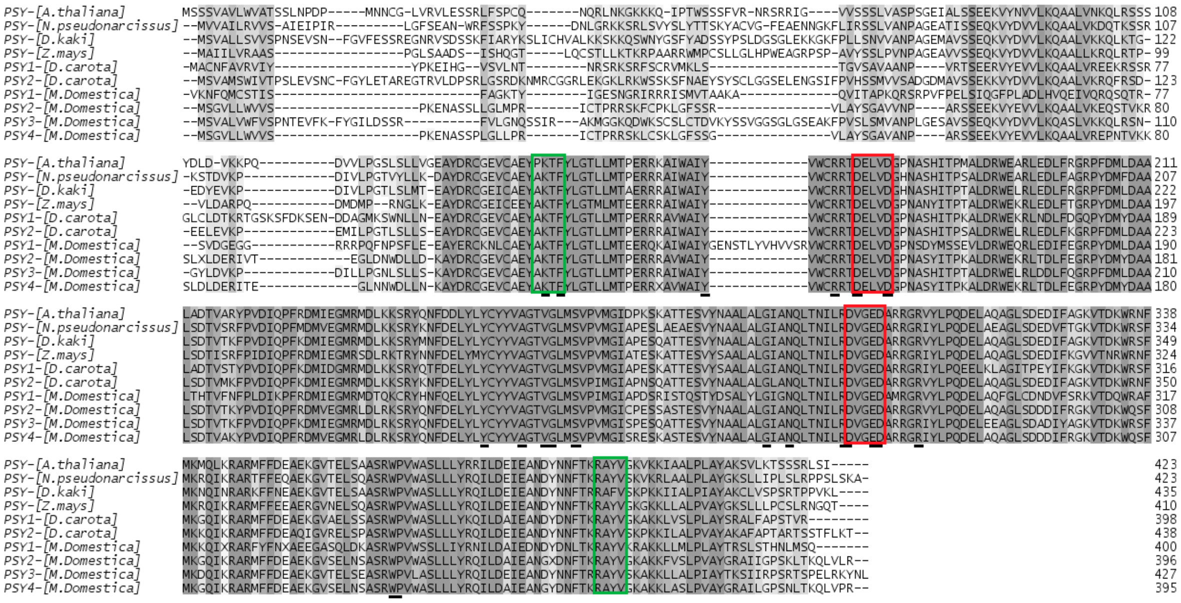

Supplementary Figure 4. Multiple alignment of amino acid sequences of different enzymes encoded by PSY genes. The figure shows the alignment of the amino acid sequence of AtPSY (GenBank accession number: AAA32836.1), NpPSY (CAA55391.1), DkPSY (ACM44688.1), ZmPSY (ACY70935.1), DcPSY1 (DQ192186.1), and DcPSY2 (DQ192187.1). In addition, the characteristic functional domain of the PSY enzymes (Trans_IPPS-HH) are indicated; the aspartate-rich regions (red rectangles), the residues that cover the active site (green rectangles) and those residues that comprise both the active site and the substrate-binding pocket (black underlined). Those residues that are most conserved among the analyzed proteins are shown on a gray background, the higher the hue of this color, the higher the degree of conservation.

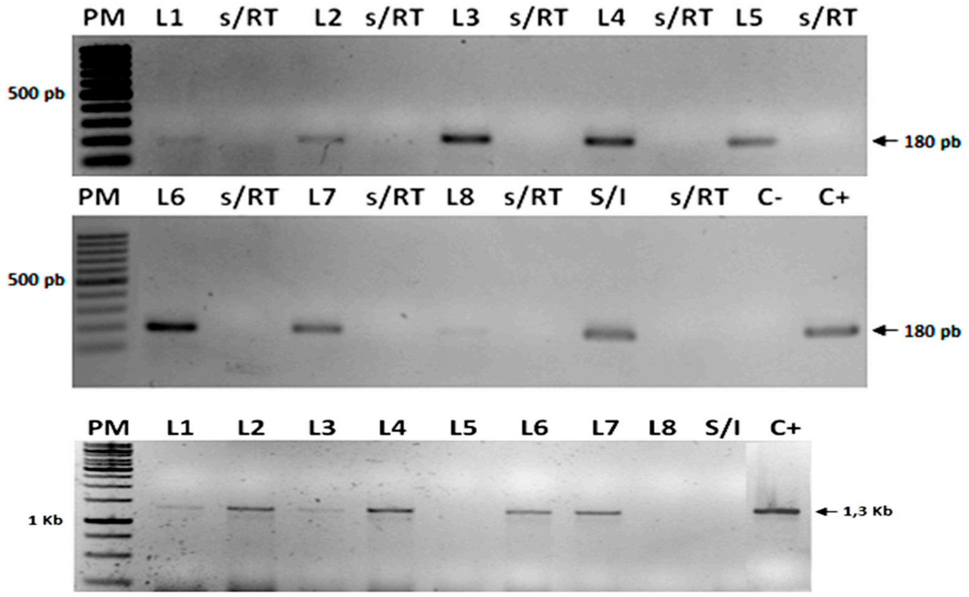

Supplementary Figure 5: Amplification by RT-PCR of the r*RNA18S* and *DcPSY2* from the transgenic *N.tabacum* lines transformed with pGWB2-DcPSY2. RT-PCR was carried out in L1-L8 for the endogenous *rRNA18S* (180bp) and the complete coding sequence of the transgene *DcPSY2* (1300bp). In the s/RT lanes, the respective controls without RT were loaded, in which mRNA from the lines analyzed was used as a template to determine the presence of gDNA in the samples. The positive control (C+) for *rRNA18S* corresponds to gDNA of wild-type *N. tabacum*. The positive control (C+) of DcPSY2 corresponds to pDNA of the pGWB2/DcPSY2 vector. S/I: amplification using cDNA from *N. tabacum* transformed with the pGWB2 empty vector (negative control). C-: Negative control, without adding DNA. MW: 1 Kb molecular weight standard. EtBr-stained 1% agarose gel electrophoresis.

Supplementary Table S1. List of primers used in this work. *DcPSY1* (DQ192186), *DcPSY2* (DQ192187), *NtPSY1* (JF461341), *NtPSY2* (JX101475), *NtLCYB* (KC484706), *NtNCED3* (JX101472.1), *NtOsmotin* (X61679.1), *RNAr18S* (AJ236016.1) and *Nt ElF1α* (AF120093.1)

Gene	Primer name	Sequence (5'→3')	Used for
UBI	ubiq-F<br>ubiq-R	GCTCGAGGACGGCAGAAC<br>CTTGGGCTTGGTGTAGGTCTTC	qRT-PCR
18S	18SF<br>18SR	TTGATTACGTCCCTGCCCTTT<br>ACAATGATCCTTCCGCAGGT	qRT-PCR
DcPSY1	psy1q-F<br>psy1q-R	AGTCGATGGAGCATTACCATAATTC<br>CTAATGGGTTACAGAGGGTTGTGTTA	qRT-PCR
DcPSY2	psy2q-F<br>psy2q-R	GCTAATAAACTTCCGTGGGTGTTC<br>GCTGGAGTTAGTGCTACCC	qRT-PCR
DcPSY2 cds	Psy2F<br>Psy2R	CGCACGTGTCATGTTTTCAG<br>AAACG GGACTAGTAATGTCAGTTGC TATGTC	Complete cds amplification, 1276 bp
NtPSY1	qNtPsy1 F<br>qNtPsy1 R	GGAACCAAGCTAATGACCCCAGAGAGA<br>TCAGAGATGTTGGAGAAGATGC	qRT-PCR
NtPSY2	qNtPsy2 F<br>qNtPsy2 R	TCAGAGATGTTGGAGA AGATGC<br>GCTTCAATCTCGTCC AATATCTTG	qRT-PCR
NtLCYB	qNtLCYB F<br>qNtLCYB R	CCGTGTTAAATTCCACCACGCCAA<br>GAAGCCAGTTGCATCAAGCACCAC	qRT-PCR
NtNCED3	qNtNCED3 F<br>qNtNCED3 R	CTTTACCAAAAACAGCCGACCCACG<br>CACCAATGGCTTTAGGAAAAACAGGACG	qRT-PCR
NtOsmotin	qNtOsmotin F<br>qNtOsmotin R	CTTCCTCCTTGCCTTGGTGACTTATACT<br>CGTGCCATTTTAGTACCTCGTGGTGCAT	qRT-PCR
Nt ElF1α	qNt ElF1a F<br>qNt ElF1a R	TGAGATGCACCACGAAGCTC<br>CCAACATTGTCACCAGGAAGTG	qRT-PCR
